# Thermal hazard evaluation on spontaneous combustion characteristics of nitrocellulose solution under different atmospheric conditions

**DOI:** 10.1038/s41598-021-03579-z

**Published:** 2021-12-15

**Authors:** Zhi-Ping Li, Jun-Cheng Jiang, An-Chi Huang, Yan Tang, Chun-Feng Miao, Juan Zhai, Chung-Fu Huang, Zhi-Xiang Xing, Chi-Min Shu

**Affiliations:** 1 School of Environmental and Safety Engineering, Changzhou University, Changzhou, 213164 Jiangsu China; 2 College of Safety Science and Engineering, Nanjing Tech University, Nanjing, 210009 Jiangsu China; 3 Department of Civil Engineering, Texas Tech University, Lubbock, TX 79409 USA; 4 School of Environmental and Chemical Engineering, Zhaoqing University, Zhaoqing, 526061 Guangdong China; 5 Department of Safety, Health, and Environmental Engineering, National Yunlin University of Science and Technology, Yunlin, 64002 Taiwan, ROC

**Keywords:** Chemistry, Energy science and technology, Engineering

## Abstract

Nitrocellulose (NC) is widely used in both military and civilian fields. Because of its high chemical sensitivity and low decomposition temperature, NC is prone to spontaneous combustion. Due to the dangerous properties of NC, it is often dissolved in other organic solvents, then stored and transported in the form of a solution. Therefore, this paper took NC solutions (NC-S) with different concentrations as research objects. Under different atmospheric conditions, a series of thermal analysis experiments and different reaction kinetic methods investigated the influence of solution concentration and oxygen concentration on NC-S’s thermal stability. The variation rules of NC-S’s thermodynamic parameters with solution and oxygen concentrations were explored. On this basis, the spontaneous combustion characteristics of NC-S under actual industrial conditions were summarized to put forward the theoretical guidance for the spontaneous combustion treatment together with the safety in production, transportation, and storage.

## Introduction

With the rapid development of modern industry, many manufactured products have entered thousands of households and been closely related to our daily lives. However, the raw materials are fraught with danger in daily life, e.g., common spray paint, coatings, plastics, artificial fibers, ink, film, and cosmetics. Nitrocellulose (cellulose nitrate, NC) is one of the raw materials of these products, which is an exceptionally hazardous nitrate ester. NC has widespread adoption in both military and civilian fields. In addition to NC with low nitrogen content (< 12.6%) mentioned above for ordinary civilian production, NC with high nitrogen content (≥ 12.6%) is used to manufacture military weapons, gunpowder, solid rocket propellants, and explosives^1–4^.

Owing to the poor thermal stability of NC, commercial NC products are wetted with solvents, or mixed with plasticizers to alleviate the risk of fire and explosion of dry NC. Universally, water or alcohols are usually added as humectants during storage and transportation to forestall spontaneous combustion. However, chemical accidents with NC still occur frequently in recent years. Among them, the most horrific was “Explosion accident in Tianjin Binhai New Area on August 12” in 2015, which led to 165 deaths, 8 missing/presumed dead, and 798 injuries^5^.

The dangerous characteristics of NC and the frequent occurrence of accidents have led numerous experts and scholars to exploring its thermal hazard since a long time ago. Some researches on the thermal decomposition mechanism of NC found that the denitrification process does not necessarily make chain breakage, because the basic structure of carbon skeleton does not change dramatically during the thermal decomposition^6^. NO_2_ generated in the thermal decomposition reaction is combined to form nitric acid groups, and then water, CO, CO_2_, carbonyl, and acid intermediates are rapidly produced^7^. Some studies obtained the critical heating rate of NC with high nitrogen content during the first order autocatalytic decomposition to thermal explosion by nonisothermal DSC technique^8–10^. Brill and Gongwer investigated the thermal decomposition characteristics of NC at different temperatures^11^. Some other researchers mainly discussed the thermal stability of NC with different particle sizes^12,13^, forms^14^, nitrogen content^15,16^, and aging periods^1^. The combustion and explosion behaviors of NC were also in focus^13,17^. In addition, a large number of experiments attached great importance to the influence of various catalysts^18,19^, stabilizers^20–24^, plasticizers^25,26^, wetting agents^2,25,27–30^, and inorganic salts^31,32^ on the thermal behaviors of NC.

As known, nitrogen is often used for protection during the transportation and storage of some hazardous chemicals. When combustion or explosion occurs, the surrounding atmosphere will also change with the reaction process^33–36^. But to sum up, although the thermal safety of NC has been extensively studied, there is still a lack of research on NC in different atmospheric conditions. Most of the existing studies focused on NC-F (F-fibre) or NC-C (C-chip), and paid less attention to the solution. However, after the Tianjin accident, the control of NC in China has become more rigorous, so it is mostly stored and transported wetted with the solution. Therefore, this paper took NC solution (NC-S) commonly used in actual production as the research object. Nonisothermal differential scanning calorimetry (DSC) and thermogravimetric analysis (TGA) technologies were applied to explore the variation rules of reaction characteristic temperature of NC-S with different concentrations in different atmospheric conditions. In order to comprehensively compare the applicability of multiple linear models for reaction kinetics calculation of NC-S, the kinetic parameters of the thermal decomposition reaction were calculated using different integral and differential kinetic models. The results revealed that several thermal stability and thermokinetic parameters of NC-S spontaneous combustion did not externalize a simple proportional relationship with the concentrations of solution and oxygen. However, the oxygen-free environment can effectively reduce the thermal risk of NC-S indeed. These findings can provide theoretical guidance for improving the treatment scheme of NC-S spontaneous combustion in actual production, transportation, and storage.

## Experiments and methods

### Materials

The NC-S sample preparation in the experiment was dissolving NC (nitrogen content less than 12.6%) in ethyl acetate (EAC). The purchased 30 mass% NC-S was diluted with EAC (content ≥ 99.5%) to obtain samples of three concentrations, which are 30, 20, and 10 mass%. The EAC sample was also used for comparison with the NC-S samples in the measurement. All dispensed samples were stored in the refrigerator at 2.0 to 6.0 °C before testing.

### Thermogravimetric analysis (TGA)

TGA is a common technique for measuring the relationship between mass and temperature^37^ by setting temperature conditions through the instrument program. The sample is placed in a specific atmosphere, and the temperature is maintained at a constant value or changed by heating scanning. By this method, the change of sample mass in the process can be observed and accurately characterized, and then the thermal decomposition characteristics of substances can be analyzed^38^.

In the experimental design, TGA 2 (produced by Mettler Toledo Co., Zurich, Switzerland) was used to test the thermal mass loss of samples. The mass (*m*) of NC-S sample with each concentration in the measurement was 7.40 ± 0.10 mg. The different heating rates (*β*): 2.0, 4.0, 6.0, 8.0, and 10.0 °C/min) were adopted respectively during the experimental temperature from 30.0 to 300.0 °C. To simulate three different atmospheric conditions, we adjusted the oxygen concentration through the gas flow meter so that the TG test was carried out at 0 vol.% oxygen (N_2_, oxygen-free environment), 10 vol.% oxygen (oxygen-lean environment), and 21 vol.% oxygen (air environment). The gas flow was 50.0 mL/min.

The mass loss, mass loss velocity, onset temperature (*T*_otg_), and peak temperature (*T*_ptg_) of three samples in different atmospheric conditions can be acquired through TG experiment.

### Differential scanning calorimetry (DSC) measurement

DSC is one of the most frequently applied thermal analysis instruments, which has extremely high sensitivity and temperature resolution and can test the weakest thermal effects. It controls temperature through the built-in program, measures the power difference in the form of heat between the input sample and reference, and obtains the relationship between heat flow and temperature. In addition, the endothermic and exothermic reaction characteristics can be analyzed by detecting the thermodynamic parameters of materials to be measured in the temperature increase condition^39^.

In this measurement, the heat-flow DSC 3 (produced by Mettler Toledo Co., Zurich, Switzerland) was applied to test the thermal decomposition behaviors of different concentrations of NC-S samples (*m* = 4.48 ± 0.08 mg) in an oxygen-free atmosphere (N_2_, 50.0 mL/min) from 30.0 to 300.0 °C^40^. We measured the heat flow change at different *β* (0.5, 1.0, 2.0, 4.0, and 8.0 °C/min) under the abovementioned conditions^41^. DSC diagram has several important thermodynamic parameters, such as peak temperature (*T*_pdsc_) and heat of reaction (Δ*H*) can be derived. Then the thermal stability of NC-S with different concentrations can also be deduced.

### Kinetic models

This study, in evaluating the difficulty of chemical reaction and reaction rate of the thermal hazardous substances, calculated the reaction kinetic apparent activation energy (*E*_a_) to study their reaction kinetics. Therefore, many dynamic models have been developed by predecessors, among which the most commonly used are some convenient model-free methods^42^. Based on previous TG and DSC experiments, the nonisothermal differential kinetic models (Kissinger, Friedman, and Starink models) and integral kinetic model (FWO model) were adopted to calculate the *E*_a_ of the spontaneous combustion reaction of NC-S. Then the effects of different atmospheric conditions and solution concentrations on *E*_a_ were summarized, and the thermal safety of NC-S was also assessed.

#### Friedman model

Several kinetic methods were derived from the following Arrhenius equations^43^ shown in Eqs. (1)‒(4):

1 $$T={T}_{\text{o}}+\beta t$$

2 $$\frac{\text{d}\alpha }{\text{d}t}=\frac{\beta \text{d}\alpha }{\text{d}T}=kf\left(\alpha \right)$$

3 $$k=Aexp(-\frac{{E}_{\text{a}}}{RT})$$

4 $$f\left(\alpha \right)={\left(1-\alpha \right)}^{n}$$

where *T* is the reaction temperature (K), and *T*_o_ is the onset temperature (K), *α* is conversion rate, *t* is the reaction time (s), *k* is the reaction rate constant, *A* is the pre-exponential factor, *R* is the universal gas constant (8.314 J/mol K). Equation (4) indicates the differential form of the kinetic mechanism function^44^.

By combining Eqs. (1)‒(4), the differential form of the first kinetic equation of thermodynamics can be obtained and shown in Eq. (5):

5 $$\frac{\text{d}\alpha }{\text{d}t}=\frac{\beta \text{d}\alpha }{\text{d}T}=Af\left(\alpha \right)exp\left(-\frac{{E}_{\text{a}}}{RT}\right)=Aexp\left(-\frac{{E}_{\text{a}}}{RT}\right){\left(1-\alpha \right)}^{n}$$

Taking the natural logarithm of both sides of Eq. (5), Eq. (6) can be acquired, which is the calculation formula of the Friedman model^45^.

6 $${\text{ln}}\frac{\text{d}\alpha }{\text{d}t}= \text{ln} \frac{\beta \text{d}\alpha }{\text{d}T}={\text{ln}}[Af\left(\alpha \right)]-\frac{{E}_{\text{a}}}{RT}$$

Friedman model is suitable for calculation with TG data, where d*α*/d*t* represents the rate of mass loss, which can be got from the derivative of mass loss to time.

#### Kissinger model

Based on the differential form of the first kinetic equation of thermodynamics, differentiate both sides of Eq. (6), Eq. (7) can further be obtained:

7 $$\frac{\text{d}}{\text{d}t}\left[\frac{\text{d}\alpha }{\text{d}t}\right]=\frac{\text{d}\alpha }{\text{d}t}\left[\frac{{E}_{\text{a}}\frac{\text{d}T}{\text{d}t}}{R{T}^{2}}-Aexp(-\frac{{E}_{\text{a}}}{RT})n{\left(1-\alpha \right)}^{n-1}\right]$$

Kissinger considered that $n{\left(1-\alpha \right)}^{n-1}$ is independent of *β*, and assumed that $n{\left(1-\alpha \right)}^{n-1}\approx$ 1. When calculated with peak temperature ($T={T}_{\text{p}}$), d/d*t*(d*α*/d*t*) = 0, Eq. (8) can be obtained:

8 $$\frac{{E}_{\text{a}}\beta }{R{{T}_{\text{p}}}^{2}}=Aexp(-\frac{{E}_{\text{a}}}{RT})$$

Taking the natural logarithm of both sides of the equation above, Kissinger model can be obtained and presented in the following Eq. (9)^37,46^:

9 $$\text{ln}\left(\frac{\beta }{{{T}_{\text{p}}}^{2}}\right)=\text{ln}\frac{AR}{{E}_{\text{a}}}-\frac{{E}_{\text{a}}}{R}\frac{1}{{T}_{\text{p}}}$$

By plotting ln(*β*/*T*_p_^2^) and 1/*T*_p_, a straight line can be fitted. *E*_a_ can be determined using the slope of the line.

#### Starink model

Starink method was adjusted from Kissinger equation, and the new Eq. (10) is as follows^47^:

10 $$\text{ln}\left(\frac{\beta }{{\text{T}}^{1.8}}\right)={C}_{\text{S}}-1.0037\frac{{E}_{\text{a}}}{R}\frac{1}{T}$$

Starink model is one of the differential kinetic methods with high accuracy and has gained widespread applications^39^.

#### Flynn–Wall–Ozawa (FWO) model

An integral kinetic model named FWO model was devised, and the formula is presented in Eq. (11)^48,49^:

11 $$\text{lg}\beta =\text{lg}\left(\frac{A{E}_{\text{a}}}{RG\left(\alpha \right)}\right)-2.315-0.4567\frac{{E}_{\text{a}}}{RT}$$

where *G*(*α*) is the integral form to the kinetic mechanism function^46^.

Plotting lg*β* and 1/*T* together in a straight line, *E*_a_ can be calculated by the slope. When FWO model is applied, only *β* and *T* are concerned, so this model is exceptionally convenient to calculate and extensively employed.

## Results and analysis

### Thermodynamic parameters of calorimetric experiments

#### Thermal thermogravimetric behaviors of NC-S

**Figure 1:**
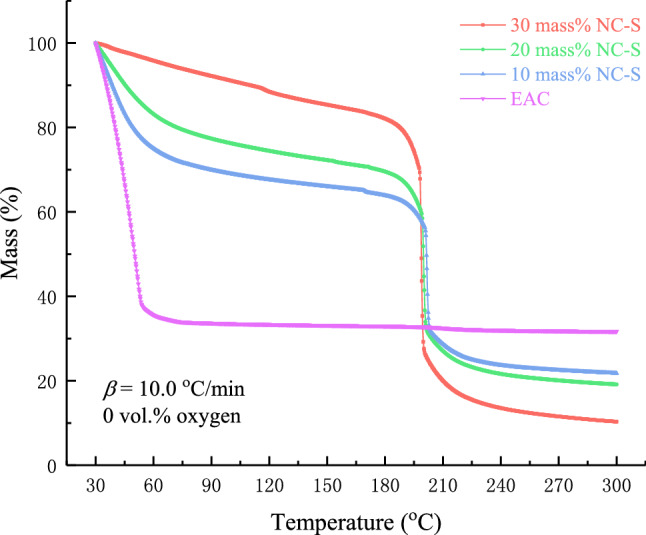
TG curves of EAC and different concentrations of NC-S in an oxygen-free environment.

Figure 1 presents the thermogravimetric loss of EAC and NC-S with three concentrations in the oxygen-free environment at *β* of 10.0 °C/min. As seen through the diagram, pure EAC experienced massive mass loss at the beginning of the measurement at 30.0 °C, and the thermogravimetric loss ended at 60.0 °C, leaving about 30% of the mass. Speculating the reason for this phenomenon is that EAC is highly volatile, and a large amount of volatilization occurred in the open-cup environment. Therefore, the thermal decomposition began with heating up. It can also be found that the thermal decomposition of NC-S occurred at about 180.0 °C, resulting in a sudden mass loss, which is related to the sample concentration. Because of EAC and NC characteristics, the initial mass loss and final residual mass of NC-S with various concentrations were different. Generally speaking, the higher content of EAC, correspondingly the lower content of NC, so the more obvious mass loss caused by EAC in the initial stage, the more residual decomposition products at the end of the heating journey, and the less mass loss caused by NC in the intermediate stage.

**Figure 2:**
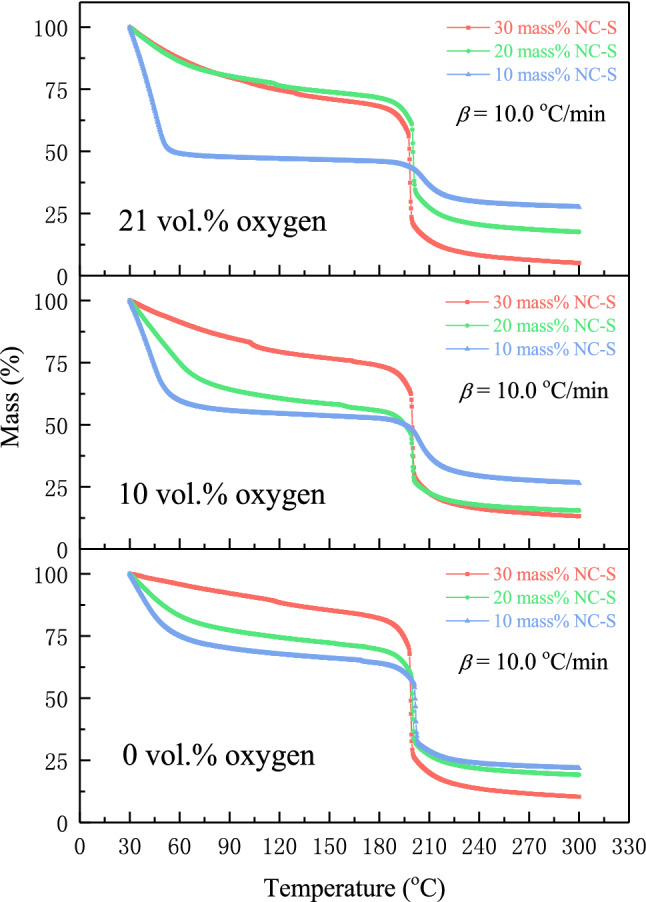
Comparison of TG curves of NC-S with different concentrations in different atmosphere conditions.

TG curves of NC-S in different atmospheric conditions at *β* of 10.0 °C/min were provided together in Fig. 2. The abovementioned rules can also be concluded by comparing the thermal decomposition thermogravimetric loss behaviors of three NC-S samples under different environments. In addition, Fig. 2 illustrates that the initial mass loss increased gradually with the improvement of oxygen concentration, which was most obvious for 10 mass% NC-S, containing the largest amount of EAC. Therefore, inferring that the oxygen concentration might greatly influence EAC’s thermal decomposition reaction at low temperatures.

**Figure 3:**
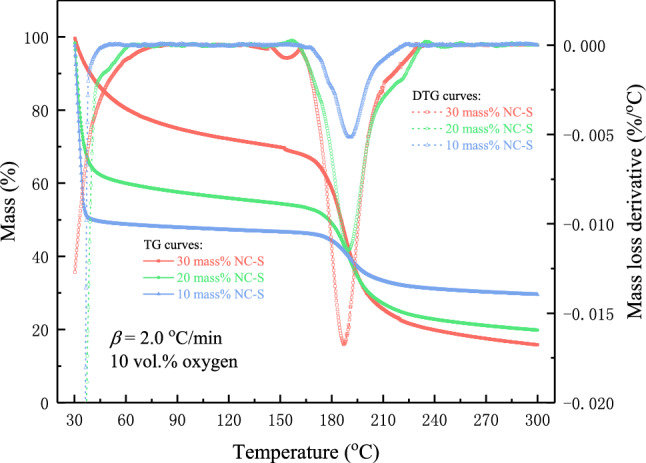
TG and DTG curves of different NC-S concentrations in an oxygen-lean environment.

TG and DTG curves of NC-S at 10 vol.% oxygen are demonstrated in Fig. 3. The TG curve indicates the mass loss process of thermal decomposition of NC-S at *β* of 2.0 °C/min. The DTG curve is the first derivative of the mass loss curve, representing the thermogravimetric loss speed of NC-S, where the peak value of DTG is the maximum rate of mass loss, and the corresponding temperature is the maximum mass loss rate temperature (*T*_ptg_). From three DTG curves, the mass loss rate of NC-S increased with the rise of solution concentration, and *T*_ptg_ slightly moved to the direction of high temperature with the decrease of solution concentration. TG curves also further confirmed the rule of thermogravimetric behavior obtained in Fig. 1. By comparing the TG curves in Figs. 1, 2 and 3, the mass of NC-S dropped smoothly at *β* of 2.0 °C/min, and when *β* was 10.0 °C/min, NC-S experienced a jump-off mass loss. Hence deducing when the temperature rises rapidly, the thermal decomposition of such a quick reaction substance would occur instantly, and the reaction is violent and hazardous.

Table 1 lists the characteristic temperatures of 30 mass% NC-S in three different environments at five *β* from 2.0 to 10.0 °C/min. As shown in Table 1, *T*_otg_ and *T*_ptg_ both increased with *β* in all kinds of atmosphere. Table 2 summarizes the average value of three NC-S samples’ characteristic temperatures and residual mass under three atmospheric conditions in TG measurement. According to the statistics data in Table 2, the oxygen concentration had little influence on the characteristic temperatures of NC-S, while as the solution concentration declined, *T*_ptg_ gradually rose. Then, by analyzing the residual mass of 30 mass% NC-S after thermal decomposition reaction, given that it was positively correlated with the oxygen concentration, considering that the participation of oxygen could make the reaction of NC-S more sufficient.

**Table 1:** Characteristic temperatures of 30 mass% NC-S measured by TG experiment at different *β*.

*β* (°C/min)	0 vol.% oxygen		10 vol.% oxygen		21 vol.% oxygen	
	*T*_otg_ (°C)	*T*_ptg_ (°C)	*T*_otg_ (°C)	*T*_ptg_ (°C)	*T*_otg_ (°C)	*T*_ptg_ (°C)
2.0	177.21	189.27	175.71	186.36	177.51	186.37
4.0	184.31	194.48	183.21	191.98	184.29	192.78
6.0	196.00	197.14	191.74	193.62	193.64	194.54
8.0	195.89	196.95	196.18	197.35	196.07	197.05
10.0	197.70	199.85	198.69	200.58	197.26	198.52

**Table 2:** Thermodynamic parameters of NC-S measured by TG experiment in different atmosphere conditions.

Sample	Atmosphere											
	0 vol.% oxygen				10 vol.% oxygen				21 vol.% oxygen			
	$\overline{m}$ (mg)	*T*_otg_ (°C)	*T*_ptg_ (°C)	Average mass remaining (%)	$\overline{m}$ (mg)	*T*_otg_ (°C)	*T*_ptg_ (°C)	Average mass remaining (%)	$\overline{m}$ (mg)	*T*_otg_ (°C)	*T*_ptg_ (°C)	Average mass remaining (%)
30 mass%	7.38	190.22 ± 9.02	195.54 ± 3.99	14.04	7.47	189.11 ± 9.53	193.98 ± 5.41	12.09	7.41	189.75 ± 8.53	193.85 ± 4.73	4.81
20 mass%	7.35	189.27 ± 8.83	196.21 ± 4.31	20.47	7.46	189.06 ± 7.29	197.08 ± 4.47	20.93	7.44	190.04 ± 8.54	195.92 ± 4.43	18.64
10 mass%	7.40	189.53 ± 8.40	198.34 ± 4.95	31.64	7.44	190.17 ± 6.81	199.64 ± 5.49	26.46	7.43	190.36 ± 7.65	199.83 ± 6.70	28.51

To conclude, the TG experimental results reveal that the thermal thermogravimetric loss of NC-S can be regarded as two stages. The first stage is the mass loss of volatilization and decomposition reaction of solvent EAC, and the second stage is the violent thermal decomposition reaction of solute NC. For the solution NC-S as a whole, the higher the concentration of the solution, the more thermogravimetric loss, and the earlier the reaction can reach the fastest speed, so there is a greater risk when the concentration of NC-S is high. By changing the experimental atmospheric conditions, the findings revealed that the oxygen concentration had little influence on the DTG temperature, but the oxygen present could affect the mass loss of EAC and make NC-S react more completely.

#### Dynamic heating examination

**Figure 4:**
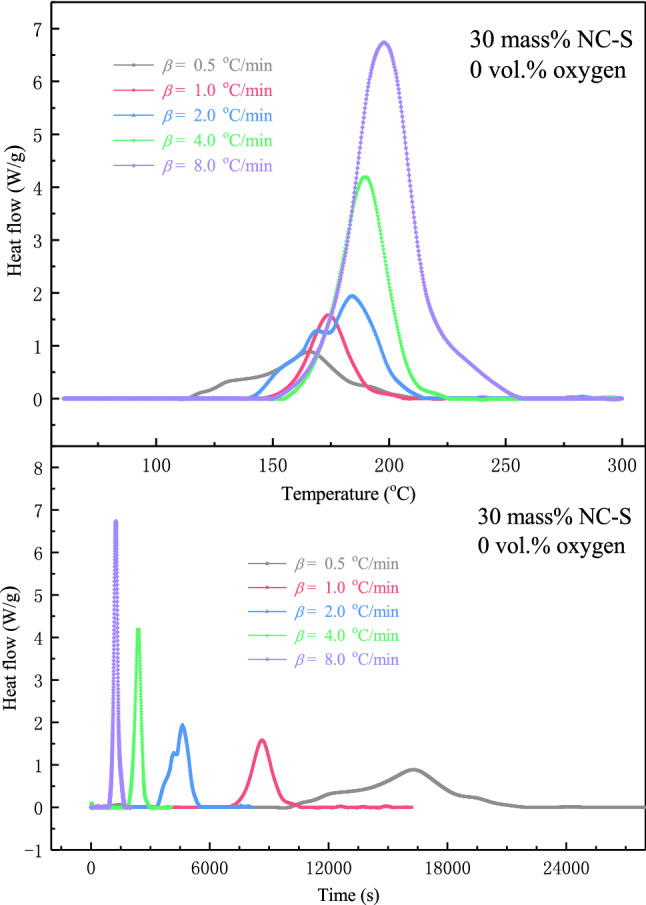
DSC exothermic curves of 30 mass% NC-S at various *β* in an oxygen-free environment.

The heat flow curves of 30 mass% NC-S at five different *β* from 0.5 to 8.0 °C/min in the DSC measurement were drawn in Fig. 4. The Y-axis value of the peak of the curve represents the maximum heat flow of NC-S in the exothermic reaction process, and the corresponding X-axis value is the temperature or time when the maximum heat flow was reached. As seen from the diagram, when *β* increased, the curves shifted to the right in the direction of high temperature, *T*_pdsc_ also moved to the direction of high temperature, and the heat flow at the peak accordingly increased. By integrating the heat flow with respect to time, Δ*H* at five different *β* can be obtained from the area of the exothermic peak. Therefore, according to the picture in the bottom part of Fig. 4, Δ*H* decreased with the *β* increased. All these findings indicate that *β* impacted the thermal decomposition of NC-S.

**Figure 5:**
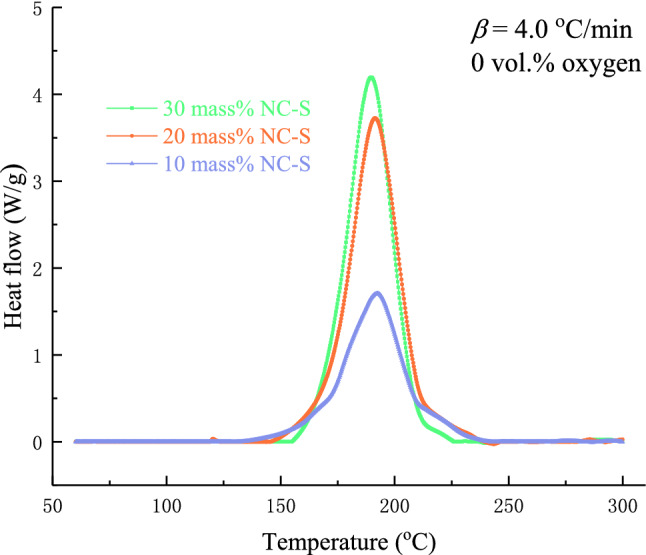
DSC exothermic curves of NC-S with different concentrations at *β* = 4.0 °C/min in an oxygen-free environment.

Figure 5 depicts the DSC exothermic curves of three concentrations of NC-S at *β* of 4.0 °C/min. The curves illustrate that *T*_pdsc_ did not change prominently with the solution concentration of NC-S, but the heat flow values varied correspondingly.

The average characteristic temperature and heat release of the three concentration samples in the DSC experiment under an anaerobic environment can be obtained from Table 3. These parameters also indicate that no correlation was between solution concentration and *T*_pdsc_. Afterward, according to the experimental data, Δ*H* of 30 mass% NC-S was 2555.55 J/g, and that of 20 mass% NC-S was 2584.55 J/g, which were similar in values and both were much higher than that of 10 mass% NC-S.

**Table 3:** Thermodynamic parameters of different NC-S concentrations measured by DSC experiment in an oxygen-free environment.

Sample	Parameter		
	$\overline{m}$ (mg)	*T*_pdsc_ (°C)	Average Δ*H* (J/g)
30 mass%	4.47	184.03 ± 13.46	2555.55
20 mass%	4.46	183.96 ± 14.58	2584.55
10 mass%	4.51	185.71 ± 12.03	1496.03

The abundant heat released by the thermal decomposition reaction can not be removed quickly, leading to heat accumulation, and the possibility of thermal runaway is enhanced. Thus, it is concluded that the higher spontaneous combustion risk is occurring in high concentration NC-S.

### Kinetic analysis

#### NC-S’s *E*_a_ calculation with TGA data

**Figure 6:**
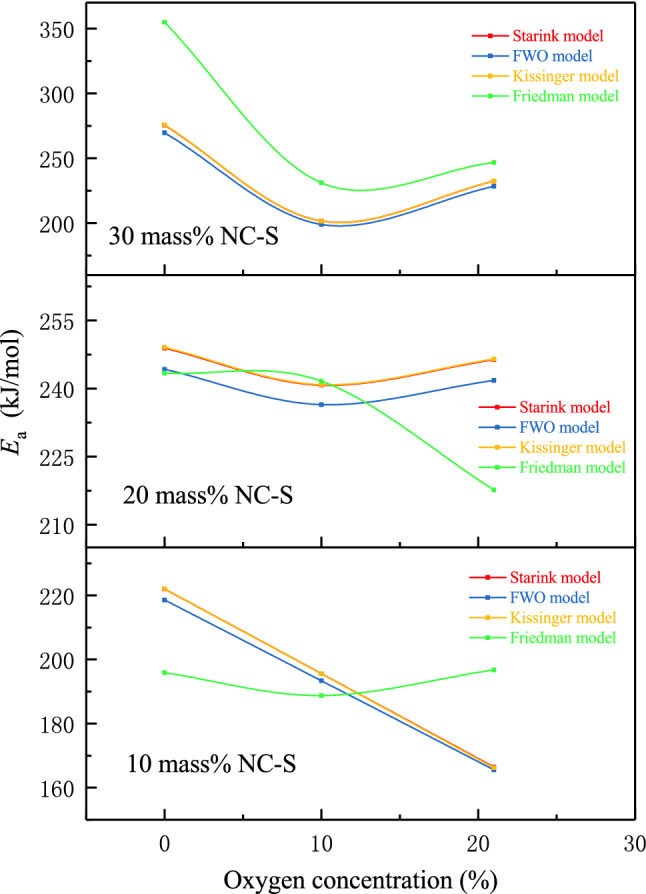
NC-S’s *E*_a_ under three different atmosphere conditions calculated by four kinetic models with TG data.

**Figure 7:**
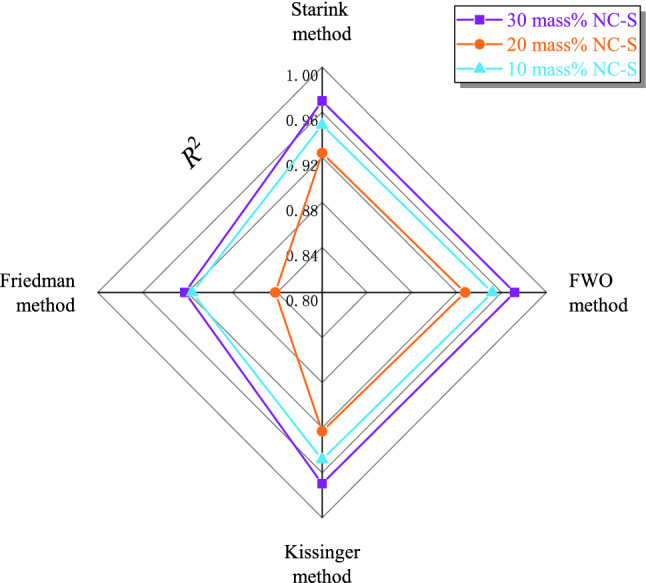
Comparison of four kinetic models’ *R*^2^ via TG data.

Based on the data measured in TG experiments, four kinetic models (Starink, FWO, Kissinger, and Friedman models) were used to calculate *E*_a_ of three samples under the different atmospheric conditions. The *E*_a_ calculated by different models were sequentially plotted as curves in order of oxygen concentration, which are given in Fig. 6. The linear regression method is usually adopted in the kinetic calculation, and the correlation coefficient (*R*^2^) is a parameter to measure the goodness of fit. Therefore, to explore the applicability of the four methods to samples, the average *R*^2^ of each model was also calculated accordingly, as shown in Fig. 7.

From Fig. 6 as a whole, no matter for which sample, *E*_a_ calculated by Starink, FWO, and Kissinger models were extremely similar in terms of the numerical values and the trends of curves changing with the oxygen concentrations. Among them, the curves of Starink and Kissinger models were even almost overlapped. However, when using the Friedman model, the calculation results were far from the other three. Analyzing combined with Fig. 7, among the four models, *R*^2^ of Friedman model was the smallest, indicating that its linear goodness of fit was the worst. Based on this analysis result, we considered that the applicability of Friedman model to NC-S is not good enough.

Figure 6 displays that *E*_a_ of 20 and 30 mass% NC-S varied with oxygen concentration in the same way, while *E*_a_ of 10 mass% sample was inconsistent with the two. Therefore, inferring that the reaction kinetics characteristics of NC-S were the same when the solution concentration was above or equal to the threshold of 20 mass%. In contrast, the thermal decomposition kinetic characteristics of the sample might display another trend when the concentration was below the threshold.

Due to the above analysis summarized that there was a large deviation in the calculation of the kinetic parameters by Friedman model for NC-S, we averaged the values calculated by the other three models (Starink, FWO, and Kissinger models) to obtain the average Ea of thermal decomposition reaction of different samples under different environments, as provided in Table 4. From the calculation results in Table 4, on the one hand, the influence of oxygen concentration can be discussed. The *E*_a_ of NC-S with any concentration was the largest in the oxygen-free environment, indicating that the thermal decomposition reaction of NC-S was relatively difficult to occur in the oxygen-free environment, and the speed of decomposition was slow. The *E*_a_ of 20 mass% and 30 mass% samples were the smallest at 10 vol.% oxygen, while *E*_a_ of 10 mass% sample decreased with the increase of oxygen involved in the reaction, and the reaction rate increased gradually. On the other hand, the influence of solution concentration can be analyzed. In the TGA experiment, 30 and 20 mass% samples obtained the maximum *E*_a_ under anaerobic and aerobic conditions, respectively. In general, *E*_a_ of 20 mass% NC-S was not saliently affected by the oxygen concentration.

**Table 4:** The average *E*_a_ calculation of NC-S in different atmosphere conditions via TG data.

Sample	Atmosphere		
	Average *E*_a_(kJ/mol)		
	0 vol.% oxygen	10 vol.% oxygen	21 vol.% oxygen
30 mass%	273.64	200.65	231.02
20 mass%	247.38	239.32	244.85
10 mass%	220.87	194.82	166.07

It was inferred from the above findings that the thermal decomposition reaction of 20 mass% NC-S was relatively stable in the aerobic environment of daily production and life, and was less affected by the changes of the external environment. In addition, 20 mass% was an ambiguous boundary, and the reaction kinetics of NC-S with a concentration above and below it presented different characteristics, which was consistent with the inferences obtained in Fig. 6.

#### NC-S’s* E*_a_ calculation with DSC data

**Figure 8:**
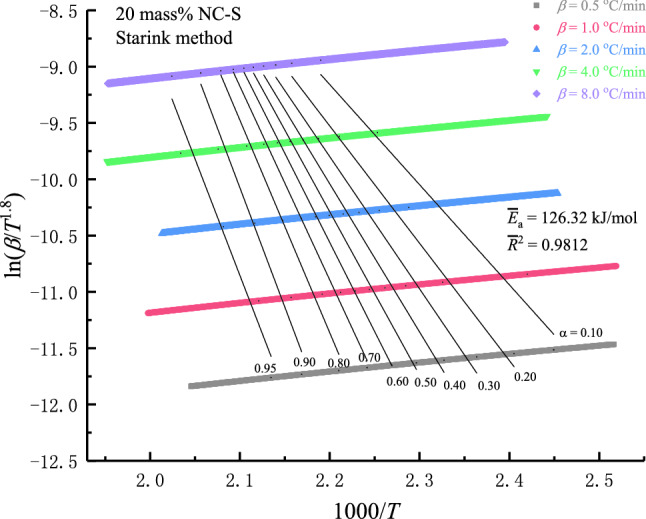
*E*_a_ fitting curves by non-isothermal differential method for *α* from 0.10 to 0.95.

Based on the data obtained from DSC measurements, *E*_a_ values of three NC-S samples in nitrogen atmosphere were calculated by nonisothermal integral method (FWO model) and nonisothermal differential method (Starink model). Figure 8 schematizes the lines obtained by fitting ln(*β*/*T*^1.8^) and 1000/*T* at different *α* via the differential model to calculate *E*_a_ of 20 mass% NC-S at different *α*. After averaging *E*_a_ at all *α*, the average value of *E*_a_ of 20 mass% NC-S in oxygen-free environment can be obtained by Starink model as 126.32 kJ/mol, and *R*^2^ was 0.9812. Correspondingly, the average *E*_a_ of 30 mass% and 10 mass% NC-S calculated by Starink model were 128.68 and 111.84 kJ/mol, respectively. The average *E*_a_ of 30 mass%, 20 mass%, and 10 mass% samples can be calculated by FWO model as 129.30, 127.05, and 113.24 kJ/mol, respectively. By comparison, it can be found that *E*_a_ acquired by these two typical integral models and differential models were extremely close. In addition, *E*_a_ of 30 mass% NC-S was the largest in the oxygen-free environment, and that of 10 mass% NC-S was the smallest.

**Figure 9:**
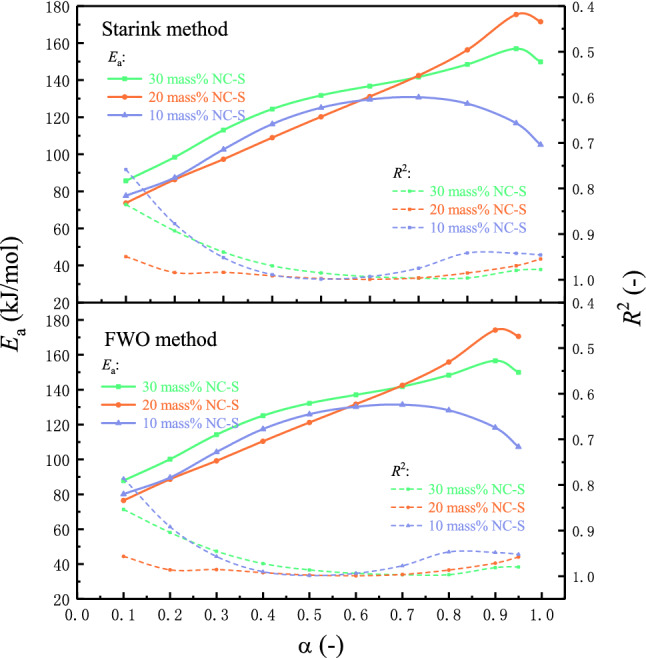
Comparison of NC-S’s *E*_a_ calculation by differential and integral kinetic models.

When *α* ranged from 0.10 to 0.95, *E*_a_ and *R*^2^ of the three samples calculated by these two models were plotted as curves, as described in Fig. 9. It is evident from the top and bottom diagrams that *E*_a_ and *R*^2^ of NC-S varied with *α* in almost the same way under these two kinetic models. Moreover, when *α* was from 0.30 to 0.95, *R*^2^ of both methods was higher than 0.90. All the above findings suggested that Starink and FWO models were exceptionally suitable for calculating the reaction kinetics of NC-S. Figure 9 also depicts that the samples with three different concentrations all had the maximum *R*^2^ when *α* was at 0.60, and the goodness of fit was the best at this time, which was also around the *T*_pdsc_. Comparing *E*_a_ of three samples at this point, 30 mass% NC-S was still the highest and 10 mass% the lowest, which confirmed the rule summarized by analyzing the average *E*_a_ before.

As a whole, the reaction kinetics analysis in the oxygen-free environment carried out with DSC experimental results further supported the previous conclusions summarized with TG data.

## Conclusions

The thermal risk and the thermal stability of NC-S with different concentrations in different environments were studied using calorimetric technology and thermal analysis. The findings are concluded as follows:

TG experiment mainly revealed the influence of solution concentration and atmospheric environment on the thermogravimetric behavior and characteristic temperature of NC-S. The entire thermal decomposition process of NC-S can be divided into two stages: thermogravimetric process of EAC and thermogravimetric process of NC. Among the three concentrations of NC-S, 30 mass% NC-S had the maximum mass loss and the smallest *T*_ptg_, which first attained the maximum decomposition speed, so 30 mass% NC-S had a greater potential thermal hazard. The thermal decomposition reaction of NC-S completed more in the air and oxygen-lean environments than in the oxygen-free environment.

DSC measurement focused on exploring the exothermic situation of the thermal decomposition reaction of three different concentrations of NC-S in the oxygen-free environment. The results indicated that *β* affected the thermal behavior of NC-S. Furthermore, once the solution concentration of NC-S was above or equal to 20 mass% in the oxygen-free environment, spontaneous thermal combustion released a large amount of heat, even more than 2500.00 J/g. If the heat were difficult to dissipate, there would be a high risk of thermal runaway after heat accumulation.

The differential and integral models were used to calculate and analyze the reaction kinetics of NC-S, respectively. Through comparison, the Friedman model was not recommended for calculating the *E*_a_ values of such a rapid reaction substance, and the goodness of fit was poor. On the other hand, the applicability of Kissinger, Starink, and FWO kinetic models to NC-S was great and could be used to calculate the reaction kinetics parameters.

According to *E*_a_ calculation results using TG and DSC data, oxygen-free environment could effectively improve the thermal safety of NC-S, the thermal decomposition reaction was the least likely to occur, and the reaction speed was the slowest. Nevertheless, in most of the actual industrial production, oxygen has always existed. At this time, 20 mass% NC-S was least impacted by the change of oxygen concentration, and the decomposition rate varied little with the amount of oxygen involved in the reaction. However, 20 mass% was also a demarcation, above or below this boundary, the kinetic parameters’ variation of NC-S with the oxygen concentration showed different rules. The low concentration of 10 mass% NC-S had the smallest *E*_a_ and was most prone to thermal decomposition under any atmospheric conditions.

In summary, if NC-S with a low concentration (10 mass%) was used in the practical industry, thermal decomposition reaction could readily occur in the air environment. However, if NC-S with a higher concentration was used, a large amount of heat would be released once the reaction took place, which was extremely dangerous. Furthermore, thermal runaway occurred with the heat accumulation, resulting in serious thermal hazards and accident consequences. As a result, to effectively reduce the potential risks of NC-S during production, transportation, and storage, the first consideration should be to create an oxygen-free environment. In this way, the probability of thermal decomposition of NC can be availably reduced, and the reaction rate can be slowed down to reduce the possibility of uncontrolled spontaneous combustion of NC-S.

For prospects, more different calorimetry instruments can be applied to explore the thermal risk of hazardous chemicals, and the mechanism function of thermal decomposition reaction can be calculated, so as to conduct a further study on its reaction kinetics.

## List of symbols

*A*: Pre-exponential factor (1/s)
*C*_s_: Constant (dimensionless)
*E*_a_: Apparent activation energy (kJ/mol)
f(α): Dynamic mechanism function of the differential form (dimensionless)
G(α): Integral form of the kinetic mechanism function (dimensionless)
*k*: Reaction rate constant (W K/m^2^)
*m*: Mass (mg)
$\overline{m}$: Average mass (mg)
*R*: Universal gas constant (8.314 J/mol K)
*R*^2^: Linear fitting’s correlation coefficient (dimensionless)
*t*: Reaction time (s)
*T*: Reaction temperature (K)
*T*_otg_: Onset temperature obtained from TG experiment (°C)
*T*_pdsc_: Peak temperature obtained from DSC measurement (°C)
*T*_ptg_: Peak temperature obtained from TG experiment (°C)
*∆H*: Heat of reaction (J/g)
*α*: Conversion rate (dimensionless)
*β*: Heating rate (°C/min)
